# Procedure of brewing alcohol as a staple food: case study of the fermented cereal liquor “*Parshot*” as a staple food in Dirashe special woreda, southern Ethiopia

**DOI:** 10.1002/fsn3.316

**Published:** 2015-11-23

**Authors:** Yui Sunano

**Affiliations:** ^1^ Graduate School of Bioagricultural SciencesNagoya UniversityFurochoChikusa‐kuNagoyaAichi464‐8601Japan

**Keywords:** Africa, brewing process, diet, fermented food, food culture, lactic fermentation

## Abstract

For most brews, alcohol fermentation and lactic fermentation take place simultaneously during the brewing process, and alcohol fermentation can progress smoothly because the propagation of various microorganisms is prevented by lactic fermentation. It is not necessary to cause lactic fermentation with a thing generated naturally and intentionally. The people living in the Dirashe area in southern Ethiopia drink three types of alcoholic beverages that are prepared from cereals. From these alcoholic beverages, *parshot* is prepared by the addition of plant leaves for lactic fermentation and *nech chaka* by adding cereal powder for lactic fermentation before alcohol fermentation. People living in the Dirashe area partake of *parshot* as part of their staple diet. The brewing process used for *parshot* and a food culture with alcoholic beverages as parts of the staple diet are rare worldwide. This article discusses the significance of using lactic fermentation before alcoholic fermentation and focuses on lactic fermentation in the brewing methods used for the three kinds of alcoholic beverages consumed in the Dirashe area. We initially observed the brewing process and obtained information about the process from the people in that area. Next, we determined the pH and analyzed the lactic acid (g/100 g) and ethanol (g/100 g) content during lactic fermentation of *parshot* and *nech chaka*; the ethyl acetate (mg/100 g) and volatile base nitrogen (mg/100 g) content during this period was also analyzed. In addition, we compared the ethanol (g/100 g) content of all three kinds of alcoholic beverages after completion of brewing. The results showed that it was possible to consume large quantities of these alcoholic beverages because of the use of lactic fermentation before alcoholic fermentation, which improved the safety and preservation characteristics of the beverages by preventing the propagation of various microorganisms, improving flavor, and controlling the alcohol level.

## Introduction

Food fermentation dates back to prehistoric times and humans have been making fermented food by exploiting the chemical action of bacteria and enzymes that exist naturally or are added deliberately (Harlander 1992). Fermentation can improve nutritional value (Simango 1997), dissolve unwanted components (Simango 1997; Sharma and Kapoor 1996), lessen the labor of food preparation (Simango 1997), and facilitate portability by reducing the volume. There are many types of fermented food in the world that are made of varied materials, using varied methods, and microorganisms. However, there are only four modes of fermentation in food processing; alcohol, lactic acid, acetic, and alkaline fermentation (Soni and Sandhu 1990). In alcohol fermentation, ethyl alcohol is produced from sugar using yeast. In lactic acid fermentation, lactic acid is produced mainly by lactic acid bacteria. In acetic fermentation, ethyl alcohol is dissolved into acetic acid and oxygen by acetobacter, and alkaline fermentation usually refers to the fermentation occurring to fish and seeds (Blandino et al. 2003). Many microorganisms are involved in these four modes of fermentation. Among these fermented foods, the most widely found are fermented beverages, a type of alcohol‐fermented food, and lactic acid‐fermented food.

Fermented alcoholic beverage has been produced since ancient times, and there are myriads of alcoholic drinks produced in this way. Roughly speaking, there are two types of fermented alcoholic beverages; a single‐step fermented alcohol that is produced by a single alcohol fermentation process, and a multistep fermented alcohol that includes saccharification as well as alcohol fermentation processes. When these fermented materials are distilled, then the beverage becomes spirits. When fermented alcoholic beverage and spirits are mixed, then it turns into liquor. Single‐step fermentation alcoholic beverages include fruit‐based drinks such as wine and cider, popular in Europe, sap‐based drinks such as palm wine, popular in Africa, Asia, and South America (Uzochukwu et al. 1994), honey‐based drinks such as mead, popular in Africa and Europe (Bekele et al. 2006), and milk‐based fermented drinks in Mongolia among others. Since these raw materials contain sugar, with naturally existing yeast, it turns into ethyl alcohol to make an alcoholic drink. On the other hand, in multistep fermentation beverages, starch in grains and tubers is saccharified first using enzymes in saliva, mold, and germinated seeds, followed by alcohol fermentation by yeast. Examples of such drinks include chicha, in which maize (Yamamoto 1995) or cassava (Mowat 1989) is saccharified by saliva, huangjiu in which grains such as rice, barley, wheat, millet and Japanese millet, or soya beans and peas are saccharified using accelerated growth of molds such as *rhizopus* and *mucor* (Yamamoto ), as well as beer in which a grain such as barley is saccharified using malt and germinated seeds. This type of alcoholic drink, especially bean sprout liquor, made using germinated seeds for saccharification is widespread around the world and in Africa, traditional local beverages using pearl millet (*pennisetum glaucum*) and sorghum are produced in many places (Oyewole ).

Another popular fermented food consumed worldwide is produced by lactic acid fermentation using mainly lactic acid bacteria. There are two types of lactic acid fermentation; homolactic fermentation produces only lactic acid while heterolactic fermentation produces carbon dioxide and ethanol in addition to lactic acid (Aguirre and Collins 1993). In both cases, any bacteria whose product is composed of over 50% lactic acid are called lactic acid bacteria (Ozaki 2009). There are several kinds of lactic acid bacteria in nature such as lactobacillus, leuconostoc, and lactococcus, and fermented foods including lactic acid‐fermented foods exploit the interaction of such bacteria as well as many other microbes (Blandino et al. 2003). Lactic acid fermentation enhances the flavor of the food (Chavan and Kadam 1989) and improves storage stability (Oyewole ). On the other hand, Western Asia is the birthplace of lactic acid‐fermented food using animal milk as raw materials, such as yoghurt, butter and cheese (Ozaki 2009). Eastern Asia is the birthplace of lactic acid‐fermented food using vegetables, such as pickles in salt and rice bran etc., sauerkraut and bread (Ozaki 2009).

Lactic acid bacteria are involved in the production process of almost all fermented food, not only in lactic acid‐fermented food (Conway 1996). Examples include kimchi, vinegar, bread, miso, and sake. Take the example of a small local brewery. Since most of such operations are carried out in nonantiseptic conditions, many microbes find their way in during the fermentation, and thus it is important to control these elements to carry out a desirable alcohol fermentation process. In most of these types of operations, while ethyl alcohol is produced by yeast from sugar, lactic acid is also made simultaneously from sugar by lactic acid bacteria which are in the environment or growing on the surface of raw materials. When lactic acid is generated, the pH of the brew decreases to under 4, which inhibits the growth of microorganisms that cause food spoilage (Daly et al., 1990), and facilitates appropriate alcohol fermentation.

Among the Dirashe people, who live in Dirashe special woreda in southern Ethiopia, lactic acid fermentation is deliberately carried out before alcohol fermentation during the production process of an alcoholic drink made of germinated grains. As described earlier, the processes of alcohol fermentation and lactic acid fermentation in small‐scale brewing operations are going naturally hand in hand, making it unnecessary to carry out deliberate lactic acid fermentation alone. The fermentation processes in this local area, both for lactic acid and alcohol, are different from any other processes in the world, in terms of fermented alcoholic drinks or preparation of lactic acid‐fermented food. Further, an “alcoholic drink”, *parshot* is given the longest lactic acid fermentation period, is considered as the “staple food”, and is thus consumed in large quantities every day. The local people obtain almost all the calories and nutrients from *parshot*. This kind of food culture is rare in the world with other few cases found in Konso in southern Ethiopia (Shinohara 2000).

As overviewed above, though various form of fermented food and drinks have been consumed in the world for millennia, the mechanisms of fermentation were elucidated only in the 1980s (Caplice and Fitzgerald 1999), thus there are many areas that need to be investigated. Identifying the preparation method of fermented foods consumed locally is important in demonstrating the diversity of the world's food culture as well as in understanding human's dietary history. On this article, focuses on the lactic acid fermentation among the above mentioned three kinds of alcoholic drink made of sorghum, describing the unique production process and discussing the significance of the lactic acid fermentation of the raw materials. The study also discusses how such a unique fermentation method and food culture was born, focusing on the interaction social and ecological elements.

## Methodology and Research Field Site

### Methodology

A total of 13 months (December 2008 to March 2009, June to August 2009, January to March 2011, January to February 2012 and February to April 2013) were spent in a village called “W” in the lower land and “Y” in the higher land in Dirashe special woreda in southern Ethiopia. Participatory observation and interviews were conducted during the stay with regard to the fermentation processes of alcoholic drinks and the diet.

The author also brought home‐frozen samples of *kalala*,* nech chaka*,* parshot*,* syuka,* which is the fermenting intermediate product of *parshot*, as well as *plota*, which is the fermenting intermediate product of *nech chaka*. As described in detail later, *syuka* is made of sorghum and maize flours by adding moringa (*moringa stenopetala*) or Ethiopian kale (*brassica carinata*) leaves. It is used after at least 2–3 months of preservation. In this study, 3‐week‐old and 3‐months old *syukas* with moringa, which is often used in “W” village, are examined as the samples. *Plota*, on the other hand, is made of sorghum and maize flour mix only, and is used after 1 month of keeping. In this study, 1‐week‐old *plota* is brought home as the sample. After arriving back to Japan, quantities of lactic acid and ethanol in grams per 100 g were determined. High‐performance liquid chromatography and gas chromatography were used for the measurements of lactic acid and ethanol, respectively. As for the *syukas*, ethyl acetate (in ppm) and volatile basic nitrogen (in mg) per 100 g were also determined. Microdiffusion analysis and absorptiometric analysis were used for determining volatile basic nitrogen and ethyl acetate, respectively.

While *kalala* was taken in its neat form among the locals, *nech chaka* was diluted twice with water and *parshot* with 1.3 times to twice with water. To prepare samples for the analyses, *kalala* was used as is, while the samples of *nech chaka* and *parshot* were diluted twice parts and 1.3 times of water, respectively. When making *nech chaka*, the yeast mash will be subjected to alcohol fermentation on the second day. It can be consumable for only 1 day after the fermentation process ends is done. On the other hand, the yeast mash will be kept 2–14 days before starting alcohol fermentation for *parshot*. It can be consumable for 3 days after preparation is completed. *Parshot* samples used in this study were those made of 2‐days old yeast mash and kept 1–3 days after completion, and made of 10‐days old yeast mash and kept 1 day after completion. The samples were frozen after stopping the fermentation by applying a water bath of 60°C for 30 min in heat‐proof containers. In order to assess alcohol concentration, ethanol per 100 g for three types of the samples was analyzed by gas chromatography after they had been brought back to Japan. All these analyses were outsourced to Japan Food Research Laboratories.

In the research field, in order to find out when lactic acid fermentation would start for *syuka* with added moringa and Ethiopian kale leaves and for *plota*, the pH of the samples were measured every day from the first day they were prepared to the 30th day of keeping, using pH meter at two villages, one in the higher land and another in the lower land. *Syuka* can be kept for 2–3 months and can be used for several months after that. So, in order to find out whether lactic acid fermentation would be still continuing during this 2–3 months period, the pH of *syuka* samples with added moringa and Ethiopian kale were measured on every month for 6 months, using pH meter. Further, in order to investigate the storage stability of the yeast mashes, the pH of yeast mashes with moringa and Ethiopian kale as well as yeast mash made only of grain flour were measured for 10 days on the research field site.

### Overview of research field site

**Figure 1:**
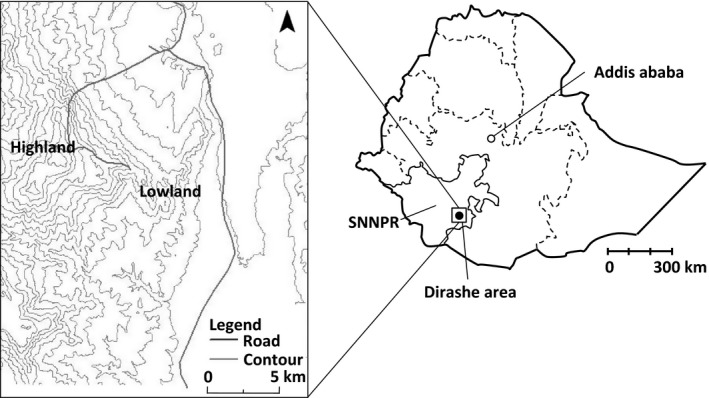
Location of research field.

The field research was conducted in a village called “W” in Dirashe special woreda in Ethiopia, which is approximately 550 km southwest of Addis Ababa (Fig. 1). According to the Health Ministry of Dirashe special woreda, the area is approximately 1500 square kilometers with a population of approximately 130,000, of which most are ethnically Dirashe people, at the time of the survey in 2008. The highest point in the area is Mount Gardolla (2561 m) with a steep slope that leads to Segen valley plateau at 1100 m above sea level. Some parts of the slope and the valley plateau where their mainstay crops of sorghum and maize are cultivated are terraced and enclosed using stones and harvest wastes. The weather is cool/cold (annual temperature ranges between 13 and 25°C) at the higher ground near the top of the mountain with year‐round rainfall. The average annual rainfall is 1300 mm. However, most areas in the woreda including “W” village are situated in semiarid land (annual temperature ranges between 17 and 31°C), with two rainy seasons in a year. With the average rainfall of only 800 mm.

The rainfall is unpredictable and unstable with large year‐to‐year differences. Good and very poor harvests alternate every few years. People in this area make storage holes in the ground called *polota* where sorghum crops can be stored for several years. They store the excess crop in a good year in preparation for a future lean year. Staple food of the people is *parshot*, a fermented alcoholic drink made of sorghum or sorghum with some maize mixed in, which have been stored in the storage holes.

## Result

### Brewing methods

**Figure 2:**
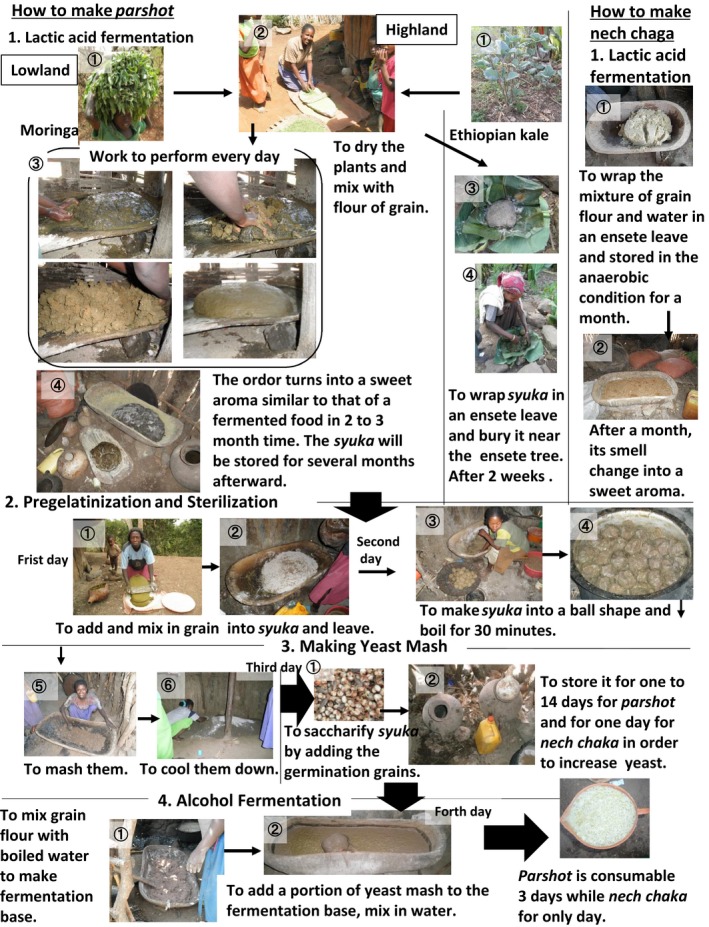
How to make alcoholic beverage.

Making *kalala* is easy; sorghum and maize flours are heated and gelatinized. Powdered germinated seeds are added to trigger saccharification and alcohol fermentation simultaneously (Fig. 2). Alcohol concentration of *kalala* is low at 1.8%. The taste is sweet and children drink it as a juice.

On the other hands, making *parshot* is more complicated and steps up to the lactic acid fermentation are different in the lower and the higher land (Fig. 2). In the lower land, a small amount of dried and powdered leaves of moringa or Ethiopian kale are added to sorghum and maize flours. The mixture is kept for 2–3 months during which lactic acid fermentation takes place until the smell of the mixture changes. Ethiopian kale is a brassica with large population of lactic acid bacteria. Moringa is also related to brassica family and can give the same, albeit inferior, effect. The mixture under lactic acid fermentation is called *syuka*. The brewer washes the surface, adds and mixes in more grain flour every day until the smell of the *syuka* changes. After 2–4 days, fruit flies (*drosophila melanogaster*) start to gather on the surface. After a further 4–6 days, *syuka* starts to emit the mixture of ammoniacal and rancid odors. If the *syuka* is left without handling, white mold starts to grow in few days. Once the smell changes, the *syuka* can be stored for several months with a monthly maintenance of washing the surface with water, adding and mixing in more grain flour, thus making it possible to make a large quantity at once and be used little by little. In the higher land, on the other hand, a small amount of Ethiopian kale powder is added to sorghum and maize flours, which will be wrapped in ensete (*ensete ventricosum*) leaves and kept in shallow holes dug near the roots of ensete plants in garden/field with several stones on top as lids and kept for 14–15 days. The steps after this stage are the same for both lower and higher lands. After adding and mixing more grain flour, *syuka* is heated to be gelatinized. Then, powdered germinated seeds of grains such as sorghum, maize, barley, and wheat are added to promote saccharification and growth of yeast. This yeast mash, with added germinated seeds, can keep up to 15 days. After that, brewing liquid made of grain flour and hot water is added to the yeast mash to start alcohol fermentation. *Parshot* is usually diluted 1.3–2 times with water to be taken and can be consumable for 3 days after the final product is made. The alcohol concentrations of *parshot*, which was made of the 2‐days old yeast mash and diluted with 1.3 parts of water just before consumption were 2.97%, 3.63%, and 3.64% on the first, second, and third day, respectively.

During the short period of very dry season of January to February in which moringa leaves are not obtainable, the people in the lower land make *nech chaka* and *kalala* from grain only to substitute *parshot* for their staple food. Leaves of moringa can be harvestable even during dry seasons, however, if the rainfall is extremely poor, it stays dormant without leaves. The production process of *nech chaka* is almost the same as that of *parshot*: sorghum and maize flour mix is kept for 1 month to undergo lactic acid fermentation until the smell changes. The mixture under lactic acid fermentation is called *plota*. As with *syuka*, the brewer repeats the process of washing the surface and adds and mixes in more grain flour every day until the smell changes. After adding and mixing more grain flour, *plota* is heated to be gelatinized (Fig. 2). Powdered germinated seeds are then added to promote saccharification and growth of yeast. At this point, in *nech chaka* making, a greater amount of germinated seeds, 1.3–1.5 times more than parashot making, is added. The local people say that unless a large amount of germinated seeds is added, fermentation does not go well. Not like *parshot*, the yeast mash of *nech chaka* does not keep and goes rotten easily, thus it has to be used all at one time. Brewing liquid is then added to the yeast mash to start alcohol fermentation, but the finished product is consumable only for 1 day. *Nech chaka* is usually diluted twice with water when consumed and its alcohol concentration is rather high at 4.10%.

It can be said that the production processes of *parshot* and *nech chaka* are divided into two; production of vegetable material pickles by lactic acid fermentation and alcoholic drink production by saccharification and alcohol fermentation. While *nech chaka* utilizes lactic acid bacteria on the grains, and in the air, *parshot* utilizes the bacteria population on the vegetable leaves. And while parshot can keep well as an intermediate product during fermentation as well as a finished product, *nech chaka* does not. Local people say, “*parshot* smells better than *nech chaka* and has more rich taste”. They also say, “*nech chaka* may sometimes upset the stomach”. They also agree that the lactic acid fermentation stage is the most important in the whole process, with comments such as “without good *syuka* or *plota*, fermentation will not proceed well and the taste will be spoiled, this is the most important”, “if you fail at the stage, the final product goes off quickly,” and “if a strange smell is mixed in at this stage, the final product may upset your stomach”. The difference in lactic acid fermentation process seems to be affecting the differences between *parshot* and *nech chaka* in terms of alcohol concentration, storage period, taste, and flavor as well as food safety. Therefore, the article hereafter focuses on the process of lactic acid fermentation.

### Maintaining low pH

**Figure 3:**
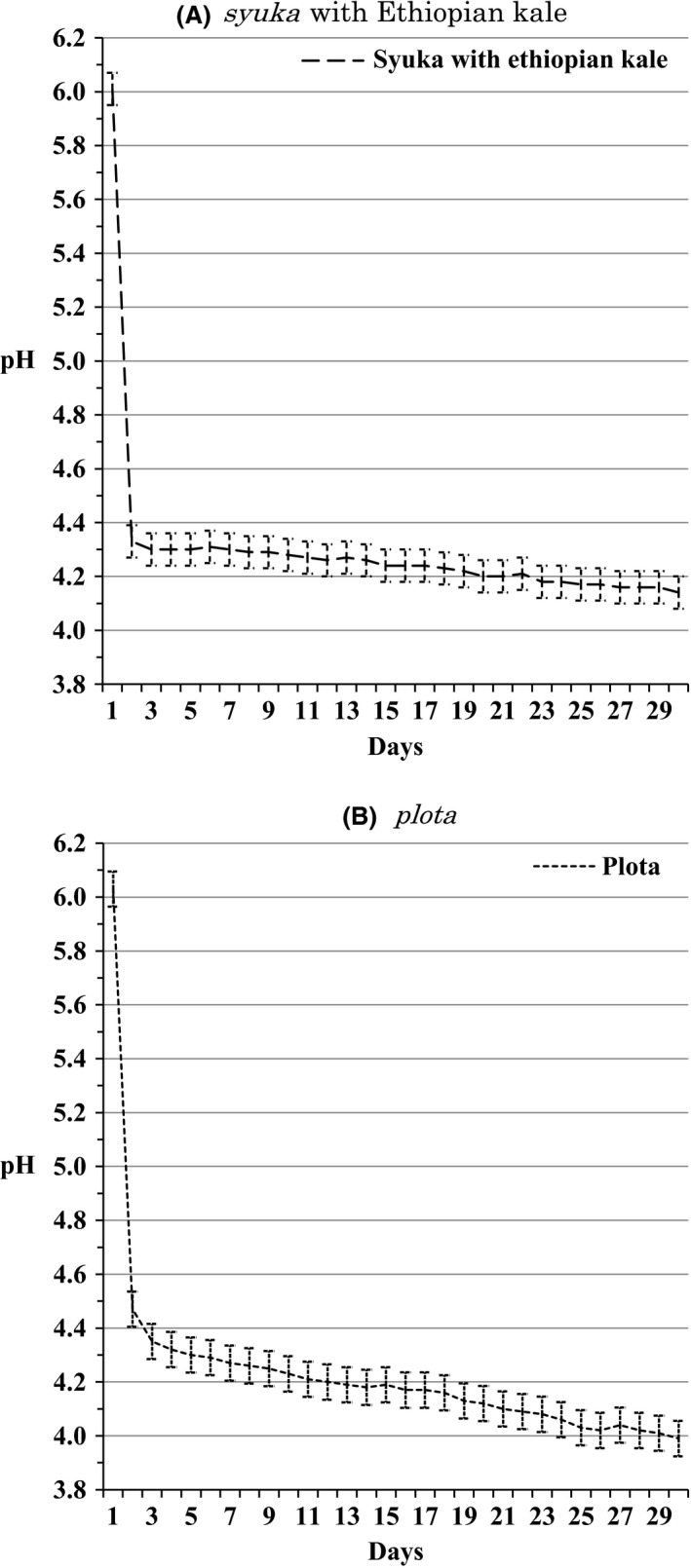
(A) pH level of syuka with Ethiopian kale produced in the highland over a month (Average ± SE Average = 4.30, SE = 0.011). (B) pH level of plota produced in the highland over a month (Average ± SE Average = 4.23, SE = 0.012).

In the higher land, the author carried out measurement of the pH of two types of syuka, one with moringa and the other with Ethiopian kale, as well as *plota* made only with grain flours, for a month. The result is shown in Figure 3. As described earlier, it takes only 14–15 days for *syuka* to be produced in the higher land. Also, in the higher land, *plota* is traditionally very rarely made. However, the people there confirm that *plota* can be generated in 14–15 days as with the syuka. The pHs of the *syukas* with moringa and Ethiopian kale went down to pH 4.33 on the second day and stayed at that level afterwards. By the 14th day, when it is said to become usable for the next step of fermentation, the pH was further reduced to pH 4.26 and pH 4.24 on the 15th day. Continuous measurement show that the reduction in the pH value continued slowly, down to pH 4.14 after 1 month. Since it has kept the pH level of 4 for a month, *syuka* can be kept for a month, however, it is usually used after 2 weeks for the next process. The pH reduction speed of *plota* is slower than *syuka* and it was pH 4.47 on the 2nd day and pH 4.35 on the 3rd day. However, the pH level afterwards did not stabilize, showing pH 4.18 on the 14th day, pH 4.19 on the 15th day, and pH 3.99 after 1 month.

**Figure 4:**
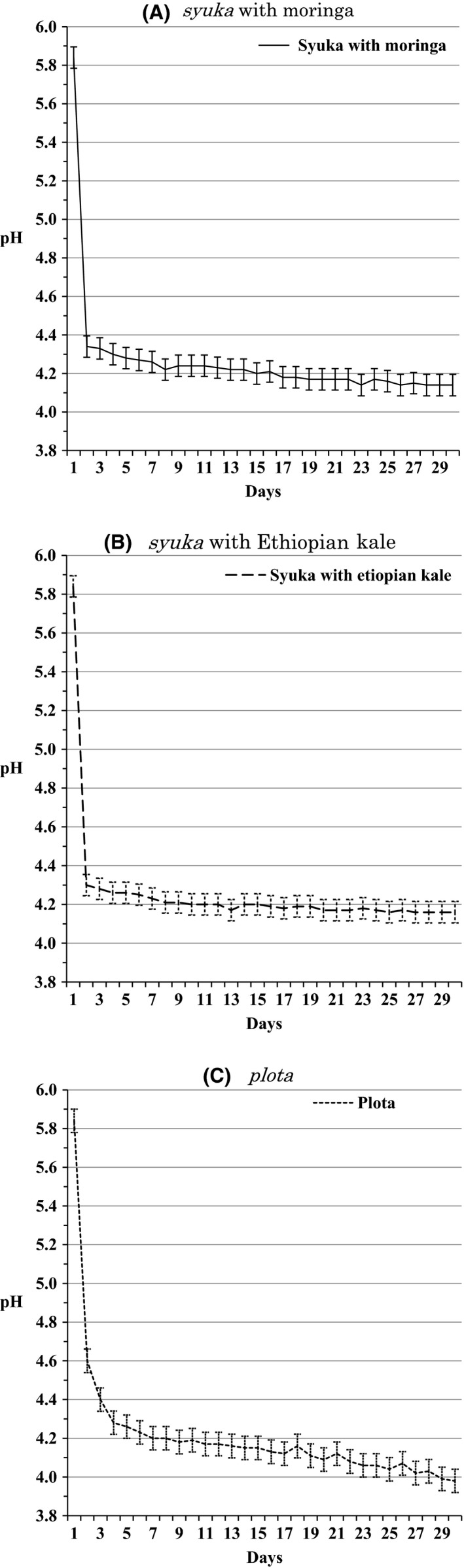
(A) pH level of syuka with moringa produced in the lowland over a month (Average ± SE Average = 4.26, SE = 0.010). (B) pH level of syuka with Ethiopian kale produced in the lowland over a month (Average ± SE Average = 4.25, SE = 0.010) (C) pH level of plota produced in the lowland over a month (Average ± SE Average = 4.21, SE = 0.011).

The other conducted the same pH measurement of two types of *syuka* and *plota*, which were in the same stage. The results are shown in Figure 4. The pH level for the *plota* did not stabilize at first. It took 4 days to be reduced to pH 4.3 and continued to decrease slowly afterwards. The *syuka*, on the other hand, reached pH 4.3 on the 2nd day and stayed at that level afterwards. Further measurements of both *syukas*, one with moringa and another with Ethiopian kale, were conducted every month for the next 6 months. The results show that both kept acidity with the pH of between 3.78 and 3.91 during that time (Table 1).

**Table 1:** pH profile of syuka and plota over 6 months

Duration of lactic acid fermentation	*Syuka*		*Plota*
	With Moringa	With Ethiopian kale	
1 month	3.91	3.88	3.89
2 months	3.90	3.87	−
3 months	3.88	3.78	−
4 months	3.79	3.90	−
5 months	3.85	3.88	−
6 months	3.91	3.85	−

The *plota*, on the other hand, reached pH 3.89 after 1 month and became suitable for the next fermentation process. However, it started to rot immediately afterwards (Fig. 4). As an experiment, the author continued to wash the surface off as well as adding and mixing grain flour into the sample of *plota*, as is the usual practice for *syuka*, even after 1 month had passed. Then, the *plota* started to emit abnormal order, gave an irritating sensation on the hand when mixing on the 34th day and started to change its color on the 36th day. At that point, the experiment was discontinued. Also, as another experiment, when another sample of *plota* had been left for longer than 30 days, it started to give off stench on the 35th day. As shown in these experiments, though *plota* changes smell and becomes usable for the next fermentation step after keeping for 1 month, the state does not last and starts to rot immediately afterwards. This is why *plota* is not kept and all of it are used as the ingredient for the next step. *Syuka*, on the other hand, can keep for several months after it has reached to the usable state, and will be used little by little for the next few months as the ingredient for the next step of fermentation.

### Lactic acid production

Since both samples of *syuka* and *plota* showed pH ranges between the upper side of 3 and the lower side of 4, it is assumed that lactic acid bacteria dominate the flora. Two types of syuka with moringa at 3‐weeks and 3‐months old and 1‐week‐old *plota*, with the pHs of which were deemed to be stable, were brought back to Japan and the volumes of lactic acid per 100 g were measured. While 1‐week‐old purota had only 1.18 g of lactic acid, 3‐weeks old *syuka* contained 4.56 g, and 3‐months old *syuka* 3.36 g of lactic acid per 100 g (Table 2).

**Table 2:** Variation in the Composition of syuka and plota at different fermentation durations per 100 g

Contained material	*Syuka*		*Plota*
	3 weeks old	3 months old	1 week old
Lactic acid (g)	4.54	3.36	1.18
Ethyl acetate (ppm)	60.00	34.00	190.00
Volatile basic nitrogen (mg)	87.00	34.00	–
Ethanol (g)	0.21	0.41	2.00

Ethiopian kale(brassica carinata) belongs to the brassica family. Moringa (*moringa stenopetala*) belongs to Moringaceae Martynov, which is closely related to brassicales. Both plants have large populations of lactic acid bacteria living on the leaves. Lactic acid bacteria growing naturally on *syuka* produce a much larger quantity of plant‐derived lactic acid compared to those growing on *plota*. Lactic acid bacteria in *syuka* increase quickly to take dominance in the flora, killing other microorganisms that produce harmful substances. However, in *plota*, lactic acid bacteria in the air and on the surface of grains take several days to dominate the flora. While lactic acid bacteria slowly build up, substances produced by other microorganisms take hold, spoiling storage quality and food safety. This is why *nech chaka* does cannot be preserved for a long time.

### Selection and proliferation of aciduric yeast under acidic condition

Not only lactic acid bacteria, but also aciduric yeast shows proliferation in *syuka* and *plota*. Two types of *syuka* of 3‐weeks and 3‐months old and 1‐week‐old *plota*, which had been prepared in the lower land, were brought back to Japan and the volumes of ethanol per 100 g were measured. In *syuka*, the amount of ethanol was found to increase as it aged, showing 0.21 g in the 3‐weeks old sample and 0.41 g in the 3‐months old sample. Ethanol in 1‐week‐old *plota* was as high as 2.00 g (Table 2). In *plota*, lactic acid bacteria come to dominate the flora on the 4th day and with that, almost all other naturally occurring wild yeasts and other microorganisms disappear. However, wild yeasts that come with the sorghum increase, prolifically producing a large amount of ethanol by the 4th day, thus a large amount of ethanol is still being generated even on the 7th day. In *syuka*, on the other hand, lactic acid bacteria come to hold a dominating position in the flora on the second day and kill off wild yeasts, thus the amount of ethanol to be generated is smaller. In both *syuka* and *plota*, once lactic acid bacteria dominate the flora, only aciduric yeasts will grow.

If *syuka* and *plota* made using the lower land method are left for several days, white mold starts to grow on the surface. This is a film yeast, which is aerobic and aciduric and grows especially where growth of other microorganisms is reduced. Thus, the growth of the film yeast on the surface of *syuka* and *plota* indicates the sufficient lowering of pH and dominance of lactic acid bacteria in the flora. The Dirashe people also see the white mold as an indicator of whether the dough becomes good *parshot* or *nech chaka*. At the same time, proliferation of the film yeast is not favored, and as soon as the growth is found, the surface of the dough is washed and folded in and mixed, creating an anaerobic condition to kill off the yeast. One of the smells emitted by the film yeast during the fermentation process is ethyl acetate. A tiny amount of ethyl acetate makes a favorable flavor, but a large amount of that smells like thinner and is not liked. Amount of ethyl acetate per 100 g in the 3‐week‐old and the 3‐month‐old syukas with moringa were 60 mg and 34 mg, respectively, showing a decrease as it aged (Table 2).

When *syuka* or *plota* is left alone, anaerobic butyric acid bacteria grow at the bottom of the dough. Though butyric acid bacterium is beneficial for humans, it emits a bad odor that reduces appetite. By mixing and sending air into *syuka* and *plota*, the local people stop the growth of the film yeast as well as the growth of butyric acid bacteria. Also, by adding grain flour when mixing, protein and sugar are provided for the lactic acid bacteria and yeasts, increasing aciduric yeast as well as reducing film yeast and butyric acid bacteria.

### Generating of flavor

Fruit flies are attracted to *syuka* within several days into fermentation, but only a small number of them are attracted to *plota* and it takes more than 1 week for them to notice it. The smell of ester compound in fermented vegetable materials attracts fruit flies (Barrows 1907), and, as described earlier, a large amount of ethyl acetate is generated in *syuka* in its early fermentation stage.

Also, around this early stage of fermentation, rancid and ammoniacal odors among other fermentation smells are emitted, very strongly from syuka and faintly from *plota*. According to interviews with 50 local people aged between 30 and 50 by the author, this bad odor “changes to a sweet fermenting smell after 2–3 months in *syuka* and after 1 month in *plota*”, and “this is the right time to move on to the next fermentation process”. The author could not recognize the change in the smell, but local people could and were using it as the indicator to start the next fermentation process. Two types of *syuka* with particularly strong smell at 3‐weeks and 3‐months old were selected as samples and brought back to Japan, and the volumes of volatile basic nitrogen per 100 g were measured. The amount of volatile basic nitrogen in the 3‐weeks old and 3‐months old *syuka* were 87 mg and 34 mg, respectively, showing a decrease as it aged, and it is assumed that rancid and ammoniacal odors that cause the bad smell has decreased accordingly (Table 2).

When grains are fermented, ethanol produced by yeasts, fatty organic acid produced by lactic acid bacteria etc., bind together to form esters that emit aromas (Imai et al. 1983; Higashi 2006). Various kinds of volatile substances are produced and mixed, then create an aroma that promotes appetite (Chavan and Kadam 1989). In *syuka*, it is thought that vegetable‐derived lactic acid bacteria break down protein and sugar and during which unpleasant ammoniacal, sulfuric, and ethyl lactate smells are emitted. However, as the resynthesis of organic and fatty acids progresses during the 3 months, the unpleasant odor changes an aroma with a hint of sweetness. It is highly likely that this sweet aroma is a mixture of fruity smells such as ethyl lactate and ethyl acetate, sulfuric compound smells such as methanethiol and dimethylsulfide (Imai et al.^1983^) and glucosinolate (mustard oil glucoside) that is the hot and bitter taste in the brassica family of plants such as wasabi and cabbage (Imai et al. 1983).

## Discussion

Fermentation has been employed as a means to improve storage quality for the last 6000 years (Wood and Holzapfel 1995). Among several types of fermentation, lactic acid fermentation plays an important role with several types of food prepared in that way. The Dirashe people make great use of lactic acid fermentation in making *parshot*, a kind of germinated grain alcoholic drink. *Parshot*, which is the product of the lactic acid fermentation, is low in contamination, maintains high food safety, and keeps well during the fermentation process as well as a finished product. Also, the lactic acid bacteria derived from the vegetable material produce a large amount of lactic acid, break down sugar and protein, resynthesize organic and fatty acids that add taste and flavor to the food. Since alcohol fermentation of *parshot* is a slow process with a small amount of germinated seeds, the alcohol concentration is low at 2.96–3.64%. When the vegetable leaves are not obtainable, *nech chaka* is made. The grain flour, minus vegetable leaves, goes through lactic fermentation, in the same way as *syuka*, for a month to produce *plota*, which is then alcohol fermented to make *nech chaka*. Lactic acid bacteria on the grains increase slowly and cannot maintain dominance in the flora over a long time with lower production of lactic acid. This makes nech chaka inferior in storage quality, food safety, and flavor. Also, it needs speedy alcohol fermentation before the contamination starts. To achieve that, a large quantity of germinated seeds is added, resulting in a higher alcohol concentration than parshot at 4.1%. In this way, the Dirashe people add green leaves that are the source of lactic acid bacteria deliberately to promote lactic acid fermentation, in order to improve storage quality, food safety, and flavor as well as making the alcohol concentration adjustable, thus it enables them to take a large amount of it every day.

But, why do they have to go through all this to take a large amount of alcoholic drink every day? In Ethiopia, there is another ethnic group of people called the Konso people whose staple diet is *chaka*, a germinated grain alcoholic drink made of sorghum. The Konso people, who live in the neighboring Konso region, descend from the Oromo ethnic group as with Dirashe, and there are commonalities in lifestyle, culture, society, and language between them. The Konso people, however, cultivate not only sorghum, maize and moringa, but also grains such as wheat and barley, tubers, legumes, and other vegetables, on stone terraces cut out of the steep hillsides (Shinohara 1998, 2000, 2002). Though they take *chaka* as the staple food twice out of four meals a day, dumplings made of tubers and grains as the staple and legumes and vegetables as secondary are also taken at the other two meals (Shinohara 2000, 2002). By this means, though the Konso people consider *chaka* brewed from sorghum and maize as their staple food, they also take nutrients from other sources.

Contrary to that, the Dirashe people rely almost entirely on the nutrients from parshot and rarely eat any other type of food. Their explanation for this is that it is difficult to grow legumes and other vegetables due to the unstable weather and serious problems of insect damage. However, the main component of sorghum and maize is starch with very little protein (Chavan and Kadam 1989). Since the nutrient values of grains improve when they are fermented (Chavan and Kadam 1989), it is assumed that these people are consuming them in alcohol‐fermented forms. Another advantage of alcohol fermentation is to convert the grains into a fluidized form, enabling the people to take a large amount without giving them a feeling of having a full stomach. It is highly possible that the Dirashe people are taking in all the nutrients necessary for everyday life from the grains, by fluidizing and fermenting, thus increasing the consumption to sustain their lives. The weather in the Dirashe region is unstable with good and very poor harvests alternating every few years. The Dirashe people keep their sorghum crop in storage holes in the ground, which are capable of keeping the crop for several years, thus securing a stable food supply every year. Though the crops do not spoil, sorghum kept in the storage holes have an unpleasant smell such as an ammoniacal odor. Explained the flavor changes when the food material is fermented (Chavan and Kadam 1989). The Dirashe people say that by making the crop into *parshot*, the unpleasant smell changes to sweet and nice aroma. They also say that lactic acid fermentation change the grains unpleasant smell, and it is assumed that lactic acid and alcohol fermentations of the grains and resulting improvement of flavor enable them to consume such stored grains. Based on these reasons, they need to have the grains alcohol fermented, as well as consume it in a large quantity. Aside from a risk of damaging the liver if taken excessively, alcohol made in a small‐scale home brewing, often in nonsterile conditions, have a possibility of contamination by other microorganisms that may spoil storage quality and food safety. The Dirashe people overcome this shortcoming by lactic acid fermentation prior to the alcohol fermentation.

However, how did they establish this kind of fermentation method? Lactic acid fermentation is a widespread form of food preparation in Ethiopia with many regions taking lactic acid‐fermented food as their staple. In the north of the country and the urban areas, a round and thin flatbread which is called injera as big as 1 m in diameter, is eaten as staple food, made of teff flour mixed with water and left for fermentation (Shigeta 2007). The lactic acid fermentation is completed in two stages (Gifawesen and Besrat 1982); lactic acid bacteria, mainly of the *enterobacteriaceae* family are used for the first fermentation for 18 h, then these lactic acid bacteria plus yeasts are used to complete the fermentation that takes 30–33 h (maximum 48 h) (Gifawesen and Besrat 1982). Injera can be preserved for 2–3 days. In the south of the country, protein stored in the pseudostem of ensete is harvested, wrapped in the ensete leaves, and left in a hole in the ground for several days to be lactic acid fermented, which will then be kneaded into a flat disk and baked into a hard bread called *kocho* (Negash and Niehof 2004; Yewelsew et al. 2006; Shigeta 2007). *Kocho* tastes mildly sour (Negash and Niehof 2004). The way *kocho* is prepared is similar to the *syuka* made in the higher land, in which grain flour with a small amount of dried leaves of Ethiopian kale and water are mixed in and kneaded, then wrapped in an ensete leaf and buried underground for 2 weeks with stones piled on top acting as a lid to make the hole anaerobic. Ensete is also grown in the higher land and *kocho* is eaten sometimes as breakfast there. Ensete is eaten widely in the highland of southern Ethiopia from the old day. Elderly peoples told that food preparation method of *kocho* was transmitted from another area to Dirashe area by flow of people. It can be assumed that the method of *syuka* making in the higher land comes from that of *kocho* making. It can also be assumed that *syuka* making in the lower land is the improved version of the one in the higher land in terms of the more efficient proliferation of lactic acid bacteria. Since the vegetable leaves are easy to obtain in the higher land, a small amount of *syuka* is made every 2 weeks on prioritizing speedier increase in lactic acid bacteria. In the lower land, however, since the vegetable leaves are scarce, the efficient increase in lactic acid bacteria from the smaller amount of leaves takes priority, thus it is made in a large quantity taking 2–3 months. As seen above, the lactic acid fermentation process in *parshot* production has its root in *kocho* making, and is completely separated from the next step, the alcohol fermentation. The alcohol fermentation stage in the production, on the other hand, has its root in a common and widespread method of alcohol making in Africa, in which starch in grains is converted to sugar by adding germinated seeds. As observed the *parshot* making, fermented food in the world is thought to have developed as a result of fermenting technologies of neighboring areas influencing each other.

## Conflict of Interest

None declared.
